# One-year follow-up of patients with hyperopia undergoing photorefractive keratectomy with Allegretto WaveLight Eye Q 400

**DOI:** 10.25122/jml-2021-0028

**Published:** 2022-04

**Authors:** Behrad Shahin, Habib Ojaghi, Firouz Amani

**Affiliations:** 1. Department of Community Medicine, Ardabil University of Medical Sciences, Ardabil, Iran; 2. Department of Surgery, Ardabil University of Medical Sciences, Ardabil, Iran

**Keywords:** hyperopia, astigmatism, PRK

## Abstract

This study aimed to examine the effectiveness of photorefractive keratectomy (PRK) in treating patients with cycloplegic hyperopia from +1.00 to +7.00 diopter using Allegretto wave Eye Q 400. This study was conducted on 25 patients with cycloplegic astigmatism ≤1 diopter and cycloplegic hyperopia between +1.00 and +7.00 diopters in 47 eyes, who successively entered into the study within 6 months and underwent PRK. Prior to PRK surgery, all the patients were examined for cycloplegic refraction (astigmatism and hyperopia), slit lamp, keratometry, fundus, and best-corrected (BCVA) and uncorrected visual acuity (UCVA) testing. These examinations were repeated after 1 week, 1 month, 3 months, 6 months, and 1 year postoperatively. The mean preop UCVA of patients was 0.76±0.28 (ranging from 0.00 to 1.3), which reached 0.19±0.22 (ranging from 0.00 to 0.78) one year after the surgery (P=0.000). There was a significant correlation between increasing astigmatism and preop cycloplegic hyperopia >5 diopters (P=0.000), corneal ring haziness at 12^th^ months (P=0.000), and 12 months cycloplegic residual hyperopia ≥2.00 diopters (P=0.000). 53.2% of the eyes (with a mean grade of 2.34) were detected with corneal ring haziness at 12^th^ months, which was significantly correlated with 12 months residual cycloplegic hyperopia of ≥2.00 diopters (P: 0.000) and cycloplegic sphere above 5 diopters (P=0.006). Although the use of photorefractive keratectomy (PRK) with Allegretto Eye Q 400 is associated with a decrease in the mean cycloplegic and improved UCVA and BCVA, its use is not recommended in cases with preop cycloplegic hyperopia above 5 diopters due to the high rate of induction of astigmatism, corneal haziness, and regression of hyperopia.

## Introduction

Visual impairments have been a major concern for the World Health Organization (WHO) in recent years [1]. Hyperopia is a common refractive error, even though its high levels are less common. High hyperopic eyes are prone to amblyopia and strabismus in childhood. In middle age, as the accommodation decreases, the need for near and far glasses becomes more pronounced [2].

Nowadays, the tendency to correct refractive errors, including hyperopia, is one of the most common reasons for visits to ophthalmology clinics. Refractive eye surgery is performed to reduce patients' dependence on spectacles or contact lenses during daily activities [3, 4].

Compared to myopic correction, hyperopia laser treatment is a more challenging process and requires an accurate diagnosis of patients' manifest refraction, integration of cycloplegic refraction, and precise centration. Hyperopic correction is performed by flattening the peripheral cornea using a ring-shaped ablation instrument to induce steepening in the central cornea, while in myopia laser correction, the central cornea is flattened directly [5, 6]. 

Another problem is that the ablation time is longer than for myopic correction, making it more sensitive to patient eye movements during surgery. Since hyperopia is more common in the elderly, presbyopia may become a problem in near fixation, causing decentration of the ablation. Excimer laser systems equipped with newer, more accurate eye trackers can overcome most of these problems. Moreover, there is a greater tendency for haze formation and regression in myopia, which may be explained by mechanical instability of corneal tissue biomechanics, postop loss of accommodative spasm, and irregular epithelial remodeling across the ablation region [6]. Clinical studies on hyperopic PRK (H-PRK) reported satisfactory effectiveness only for the cases with a correction of up to +4.5 diopters (D). However, H-LASIK is performed according to its Food and Drug Administration (FDA) approval to correct hyperopia up to +6.00 diopters of spherical equivalent (SE) [7, 8]. More recent studies showed that LASIK could be used to correct hyperopia as high as +7.00 D. Although results of hyperopic corrections are encouraging, predictability and safety have been poorer than myopic treatments [9, 10].

The factors decreasing the success of laser surgery in the correction of hyperopia include a small optical zone that causes corneal irregularities, loss of BCVA, regression, induced astigmatism, and poor quality of vision due to induction of higher-order aberrations [2, 11].

In recent years, flying spot lasers have improved the correction quality of hyperopia and astigmatism [12]. Allegretto-wave excimer laser is a flying spot laser that works on a 0.95 mm Gaussian spot with a frequency of 200 Hz. This laser is combined with a video system with a frequency of 250 Hz to compensate for eye movements [10, 12]. Despite the many studies on the success of PRK and LASIK in the treatment of hyperopia even at high levels with various devices such as ALLEGRETTO in recent years [3–6, 8, 13], the experience of the corresponding author of the present study in higher levels of hyperopia, and the presence of numerous contradictory papers on the results of PRK in the correction of hyperopia, the present study aimed to examine the effectiveness of photorefractive keratectomy (PRK) in treating patients with cycloplegic hyperopia from +1.00 to +7.00 diopter using Allegretto wave Eye Q 400. The study was performed prospectively by a single surgeon (Ojaghi H) at Noor Surgery Center in Ardabil (North West of Iran).

## Material and Methods

The present study is a prospective interventional consecutive case study in which 25 patients with cycloplegic astigmatism ≤1 diopter and cycloplegic hyperopia between +1.00 and +7.00 diopters in 47 eyes were successively included in the study within 6 months and underwent PRK. 

Patients with the following criteria were included in the study: (1) 18 years of age or over at the time of surgery, (2) stable refraction with a change <0.5 D in hyperopia or cylinder in the past 6 months, and (3) the ability to continue with examinations for at least 12 months postoperatively.

Exclusion criteria were as follows: (1) patients with suspected keratoconus or ectasia, (2) notable cataract or retinal pathology, (3) active ocular disease, *e.g.*, glaucoma, uveitis, severe dry eye or blepharitis, (4) patients with a history of herpes simplex or herpes zoster, (5) 12 months postop K reading of more than 50, (6) cycloplegic-subjective difference of refraction >2 D, (7) corneal warpage (*i.e.*, topographical abnormalities induced by contact lens using), and (8) thin cornea (<480 μm or expected postoperative residual stromal thickness <300 μm). In addition to the above, patients with certain conditions and disorders (*e.g.*, pregnancy, lactation, diabetes, connective tissue disease, severe atopy, and immunocompromised state), those who participated in a clinical trial using another ophthalmic drug or device, and those who were sensitive to concomitant medications were also excluded from the study [6, 11, 12, 14–16]. 

A comprehensive preoperative evaluation of the patients with the criteria mentioned above was performed, including a history, slit-lamp biomicroscopy, Air-puff tonometry (CANON, Full Auto Tonometer TX-20P, Tokyo, Japan), dilated fundus examination, manifest and cycloplegic spherical and cylindrical refraction, visual acuity testing with and without correction and upper mentioned tests repeated postoperatively during 4 consecutive FU examinations (1 month, 3 months, 6 months and 12 months after surgery).

Scheimpflug topography device (pentacem HR; Oculus, Wetzlar, Germany) was used to obtain corneal topographies. The refraction acquisition process was performed pre-and postoperatively by one ophthalmologist and the same refractometer (CANON, RK-F2 Full Auto Ref-keratometer, Tokyo, Japan), using visual acuity tables following standardized protocols.

All surgeries were performed at Ardabil (northwest of Iran) Noor Eye Laser center (a private practice setup) by a single surgeon (Ojaghi H).

Cycloplegic objective refraction was performed half an hour after instillation of cyclopentolate drop in the eyes twice 5 minutes apart, and then the eyes were examined with an indirect ophthalmoscope. Given the possibility of ocular cyclotorsion, the cornea of all patients was marked at 6 and 12 o'clock using a gentian violet marker before the surgery. After informed consent was collected from the patients, they were subjected to the surgery using WaveLight Allegretto Wave Eye-Q-400 Hz Excimer Laser (Erlangen, Germany) at an ablation zone, transition zone, and optical zone of 9, 1.25, and 6.5 mm, respectively.

In all cases, 0.02% mitomycin C (MMC) was applied for 1 minute, focusing mainly on the periphery of the ablation zone. Because hyperopia tends to progress with age, regress after PRK, and become more symptomatic with developing presbyopia, we treated our patients with full cycloplegic refractive error [10].

Postoperative treatment of the patients included the use of betamethasone 0.1% drops (Sina Darou Laboratories, Iran) with a starting dose of one drop every 2 hours and a gradual decrease over 1.5 months, levofloxacin 0.5% drops (SANTENOY, Finland) in a dose of one drop every 4 hours during the first 10 days, Artelac 0.2% drops (BAUSCH & LOMB, France) with a starting dose of one drop every 4 hours for 1 month and decreasing to one drop every 6 hours for 2 months, and dicloptin 0.1% drops (Sina Darou Laboratories, Iran) in a dose of one drop every 6 hours for the first 2 days of the surgery. Patients were visited and examined periodically after 30 minutes, one week, one month, 3, 6, and 12 months postoperatively. At each visit, the patients underwent refraction and visual acuity testing.

The patients were divided into 2 groups according to cycloplegic hyperopia:

• Hyperopia ≤5.00 D;
• Hyperopia >5.00 D.

The procedure proposed by Fantes *et al.* [11, 13, 17] was used to evaluate the corneal haze grading in patients (from 0 – no haze to 4 – details of the lens and iris not discernible).

### Statistical analysis

The results were analyzed using SPSS version 25 for windows (SPSS, Inc) and indicated as mean±standard deviation. Student's t-test was used to determine statistically significant differences between meanings, and a paired sample t-test for equally distributed data. The results of visual acuity were represented in LogMAR values. Moreover, ANOVA was used to compare the study variables between the groups. A P-value ≤0.05 was regarded as statistically significant. The safety index of the hyperopic PRK surgery was described as the mean postoperative CDVA divided by the mean preoperative CDVA, while the efficacy index was the mean postoperative UDVA divided by preoperative CDVA. The number of eyes within the intended refraction±0.50 D was defined as predictability. Refractive stability was described as a change of ±0.50 D or less in the refraction sphere, mean keratometry, or SE between follow-ups [9, 16, 18].

## Results

The present study was performed on 47 eyes of 25 patients, including 16 males (64%) and 9 females (36%), with a mean age of 33.42±10.5 and the age range of 18–52 years.

**Figure 1.**
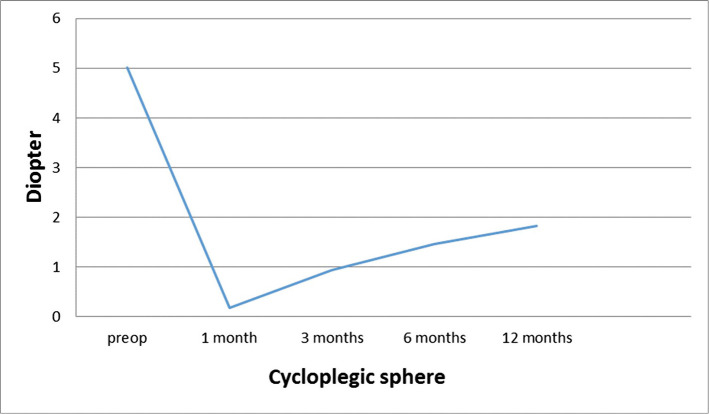
Cycloplegic sphere over time.

As shown in Table 1, there is a significant decrease and hyperopic correction in the cycloplegic sphere 1 month after the surgery compared to the preop cycloplegic sphere. However, in the subsequent visits, there was a regression of 0.18±0.91 D in the 1^st^ month to 1.83±1.72 D in the 12^th^ month (Figure 1). At 6 months after the surgery, the regression was more than 0.5 D, though between 6 and 12 months, it was approximately 0.36 D, indicating the stability of the refraction at the 6^th^ month after the surgery.

**Table 1.** Cycloplegic sphere and spherical equivalent (SE) over time.

**Parameter**	**Mean±SD**	**Range**	**Mean diff±SD**	**P**
**Sphere (D)**				
Preop *vs.* 1 month	5.02±1.64 0.18±0.91	1.50–7.00 -1.50–2.05	4.84±1.37	0.00
1 month *vs.* 3 month	0.18±0.91 0.94±1.16	-1.50–2.50 -1.25–4.00	-0.76±0.41	0.00
3 months *vs.* 6 months	0.94±1.16 1.46±1.49	-1.25–4.00 0.75–5.25	0.52±0.51	0.00
6 months *vs.* 12 months	1.46±1.49 1.83±1.72	0.75–5.25 -0.75–5.75	-0.36±4.6	0.00
12 months *vs.* Pre-op	1.83±1.72 5.02±0.64	-0.75–5.75 1.50–7.00	-3.19±1.51	0.00
**Spherical Equivalent (D)**				
*Pre-op* *vs.* *1 month*	4.76±1.55 -0.24±0.87	1.38–6.50 -2.13–2.00	5.00±1.41	0.00
*1 month* *vs.* *3 months*	-0.24±0.87 0.55±1.03	-2.13–2.00 -1.13–3.38	0.8±0.41	0.00
*3 month* *vs.* *6 months*	0.55±1.03 0.99±1.31	-1.13–3.38 -0.75–4.25	0.43±0.46	0.00
*6 months* *vs.* *12 months*	0.99±1.31 1.35±1.51	-0.75–4.25 -1.00–5.38	0.35±0.36	0.00
*12 months* *vs.* *Pre-op*	1.35±1.51 4.76±1.55	-1.00–5.38 1.38–6.50	-3.41±1.56	0.00

SD – Standard Deviation; D – Diopter; diff – Difference.

**Figure 2.**
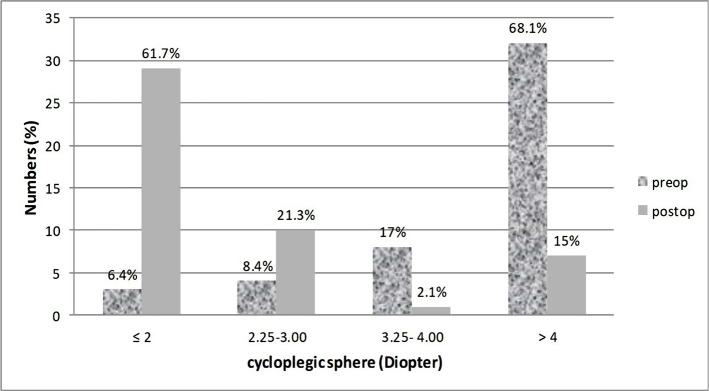
Comparison of preoperative and postoperative (12 months) cycloplegic sphere.

Compared to the preop cycloplegic sphere, the postop (12 months) cycloplegic sphere had a significant decrease so that only 3 eyes (6.4%) had hyperopia of ≤2 D preoperatively, which increased to 29 eyes (61.7%) at the12 months postop (Figure 2).

Seven (14.9%) and 15 (31.9%) eyes had a cycloplegic sphere of ≤3 and ≤4, respectively, which increased to 39 (83%) and 49 (85.1%) eyes at the 12 months postop.

Seven eyes (14.9%) had cycloplegic spheres of >4 postoperatively (12 months), which was observed in 32 eyes (68.1%) preoperatively.

**Figure 3.**
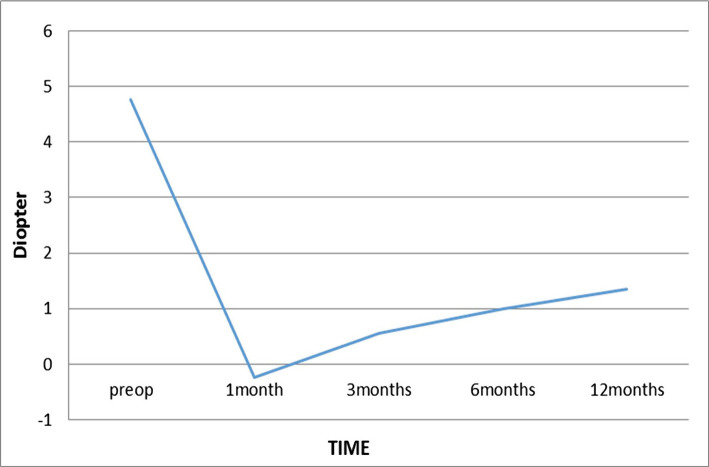
Cycloplegic spherical equivalent over time.

As shown in Table 1, there is a significant decrease in the mean of spherical equivalent one month after PRK (0.24±0.87) compared to preoperation (4.76±1.55) (P=0.00). At the 3 months postop visit, there was a regression and recurrent hyperopia of 0.8±0.41 D, and the regression continued until 12 months postop. However, due to the regression rate at the 6 and 12 months postop (less than 0.5 D per visit), it can be concluded that the spherical equivalent refraction at the 3 months postop has reached stability, though continued at a lower rate until 12 months postop (Figure 3).

**Figure 4.**
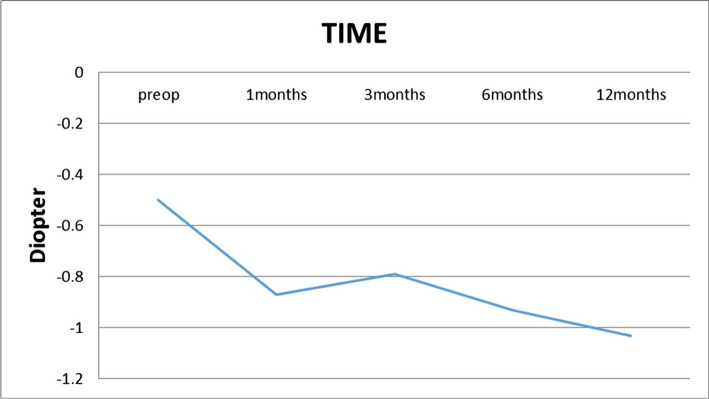
Cycloplegic mean astigmatism over time.

**Figure 5.**
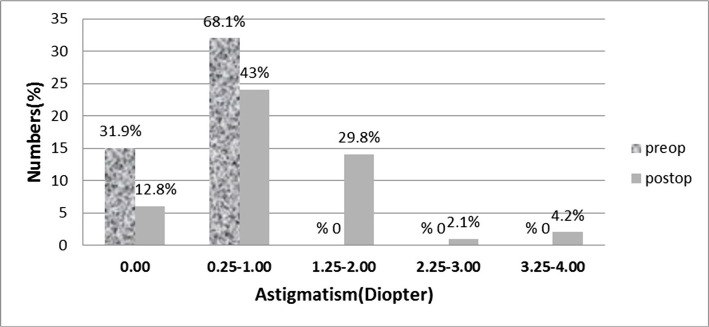
Comparison of preoperative and postoperative (12 months) cycloplegic astigmatism.

As shown in Table 2, there is a significant increase (P=0.00) in mean astigmatism at the 12 months postop (-1.03±0.88) compared to preop (-0.5±0.41). Also, there was no case of astigmatism >1 D in the preop examination, though 17 eyes (36.2%) had astigmatism of >1 D (1.25–3.75) 12 months postoperatively (Figures 4 and 5).

**Table 2.** Cycloplegic refractive astigmatism and mean keratometry (K) over time.

**Parameter**	**Mean±SD**	**Range**	**Mean diff±SD**	**P**
**Astigmatism (D)**				
*Pre-op* *vs.* *1 month*	-0.50±0.41 -0.87±0.44	-1.00–0.00 -2.00–0.00	0.36±0.48	0.00
*1 month* *vs.* *3 month*	-0.87±0.44 -0.79±0.51	-2.00–0.00 -2.50–0.00	0.07±0.42	0.2
*3 months* *vs.* *6 months*	-0.79±0.51 -0.93±0.71	-2.50–0.00 -3.00–0.00	-0.14±0.35	0.008
*6 months* *vs.* *12 months*	-0.93±0.71 -1.03±0.88	-3.00–0.00 -3.75–0.00	-0.09±0.26	0.01
*12 months* *vs.* *Pre-op*	-1.03±0.88 -0.50±0.41	-3.75–0.00 -1.00–0.00	-0.52±0.83	0.00
**Mean K (D)**				
*Pre-op* *vs.* *1 month*	42.65±1.26 47.03±2.06	39.50–45.31 42.25–51.00	-4.37±1.52	0.00
*1 month* *vs.* *3 months*	47.03±2.06 46.40±1.97	42.25–51.00 42.00–50.25	-0.62±0.65	0.00
*3 month* *vs.* *6 months*	46.40±1.97 45.87±2.02	42.00–50.25 42.25–49.88	-0.52±0.46	0.00
*6 months* *vs.* *12 months*	45.87±2.02 45.48±2.13	42.25–49.88 41.50–49.75	-0.38±0.47	0.00
*12 months* *vs.* *Pre-op*	45.48±2.13 42.65±1.26	41.50–49.75 39.50–45.31	2.83±1.38	0.00

SD – Standard Deviation; D – Diopter; diff – Difference.

**Figure 6.**
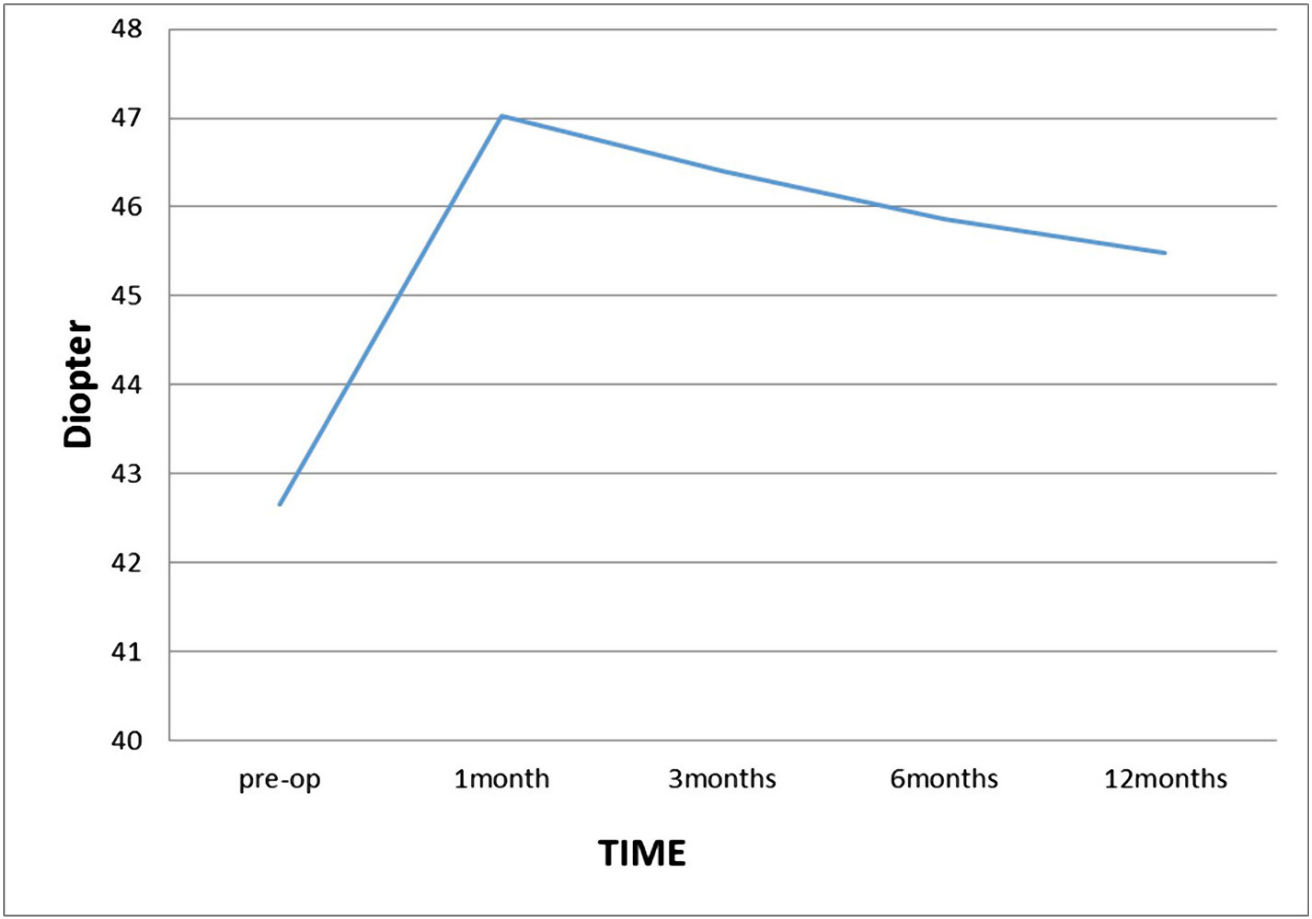
Mean keratometry over time.

Table 2 also shows a significant increase in the mean keratometry at 1 month postop compared to preop, and a relative decrease in the mean keratometry in subsequent visits at 12 months postop. The change in mean keratometry at 6 and 12 months was >0.5 and 0.38 D, indicating the stability of the mean keratometry at the 6 months postop (Figure 6).

Table 3 shows that the incidence of astigmatism >1 D at the 12^th^-month postop was significantly higher in the patients with preop cycloplegic hyperopia of >5 D than in the patients with preop cycloplegic hyperopia <5 D.

**Table 3.** Correlation of postoperative (12 months) residual astigmatism with other parameters.

**Parameter**	**Astigmatism≤1.00 D** **n (%)**	**Astigmatism>1.00 D** **n (%)**	**P**
**Pre-op cycloplegic Hyperopia (D)**			
≤5.00	19/20 (95%)	1/20 (5%)	0.00
*>5.00*	11/27 (40.7%)	16/27 (59.3%)	0.00
**Corneal Ring Haziness (G: 0.5–4)**			
*Positive*	9/25 (36%)	16/25 (64%)	0.00
*Negative*	21/22 (95.5%)	1/22 (4.5%)	0.00
**Post-op (12 months) Residual Hyperopia (D)**			
*<2.00*	24/27 (88.9%)	3/27 (11.1%)	0.00
≥2.00	6/20 (30%)	14/20 (70%)	0.00

D – Diopter; G – Grading.

Also, even though none of the patients had astigmatism of >1 D at the preop visit, out of 47 eyes, 17 had an induction of astigmatism >1 D, and it was significantly high in the patients with residual cycloplegic hyperopia ≥2 D at the 12 months postop.

Although induction of astigmatism >1 D (P=0.047) and residual cycloplegic hyperopia ≥2 D at the 12 months postop (P=0.036) was significantly higher in patients ≤35 years than >35 years, this can be justified by the fact that the number of patients ≤35 years of age with hyperopia >5 D (P=0.001) was significantly more than the patients >35 years of age.

As shown in Table 4, the corneal haziness is low in the first months, but from the 6 months postop increases, and there are 25 eyes (53.2%) with a mean haziness of 2.34 at the 12 months postop.

**Table 4.** Postoperative corneal haziness over time.

**Corneal haziness** **(G: 0.5–4.00)**	**1 month n (%)**	**3 months n (%)**	**6 months n (%)**	**12 months n (%)**
**Trace (0.5)**	0.00 (0.00)	0.00 (0.00)	1 (2.1%)	1 (2.1%)
**Minimal (1.00)**	0.00 (0.00)	1 (2.1%)	2 (4.3%)	2 (4.3%)
**Mild (2.00)**	0.00 (0.00)	4 (5.8%)	13 (27.7%)	13 (27.7%)
**Moderate (3.00)**	0.00 (0.00)	0.00 (0.00)	8 (17%)	6 (12.8%)
**Severe (4.00)**	0.00 (0.00)	0.00 (0.00)	1 (2.1%)	3 (6.4%)
**Total**	0.00 (0.00)	5 (10.6%)	25 (53.2%)	25 (53.2%)

G – Grading; n – number.

Table 5 shows that the incidence of haziness at the 12 months postop in the eyes with preop cycloplegic spheres >5 D was significantly higher than in the eyes with cycloplegic spheres ≤5 D.

**Table 5.** Correlation of postoperative (12 months) corneal ring haziness with cycloplegic hyperopia.

**Parameter**	**Corneal haziness (-) n (%)**	**Corneal haziness (+) n (%)**	**P**
**Preop cycloplegic Hyperopia (D)**			
≤5.00	14/20 (70%)	6/20 (30%)	0.006
*>5.00*	6/27 (29.6%)	19/27 (70.4%)	
**Postoperative (12 months) Residual Hyperopia (D)**			
*<2.00*	19/27 (70.4%)	8/27 (29.6%)	0.00
*≥2.00*	3/20 (15%)	17/20 (85%)	

D – Diopter; n – number.

Also, out of 27 eyes with postop residual cycloplegic hyperopia <2 D, 8 eyes had corneal haziness (0.5–4 grade) whereas, out of 20 eyes with postop residual cycloplegic hyperopia ≥2 D, 17 eyes had corneal haziness, indicating the high incidence of haziness in the eyes with residual hyperopia ≥2 D. Also the incidence of astigmatism >1 D was significantly higher in the eyes with corneal haziness (0.5–4 grade) than the eyes with no haziness at the 12 months postop.

Although the incidence of haziness at the 12 months postop was higher in patients <35 years (17 eyes out of 27 eyes: 63%) than >35 years (8 eyes out of 20 eyes: 40%), this difference was not significant (P=0.119).

At the 12 month postop, out of 47 eyes, 15 (31.9%) had an iron deposition ring around the optical zone, and there was no significant difference between the two groups of hyperopia >5 and ≤5 D regarding the iron deposition (P=0.8).

**Figure 7.**
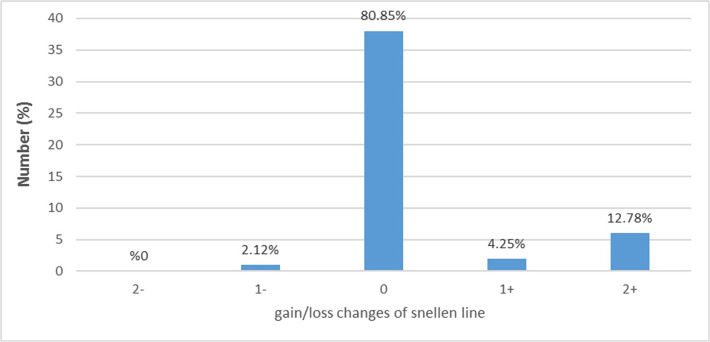
Difference between 12 months postoperative and preoperative BCVA (Snellen Lines).

**Figure 8.**
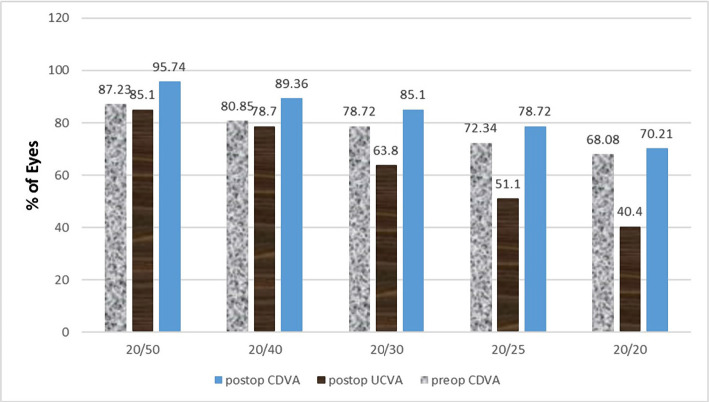
Cumulative Snellen visual acuity.

As shown in Table 6, both postoperative UCVA (P=0.00) and BCVA (P=0.01) were significantly higher than in preoperation. In the preoperative examination, 2.1%, 10.6%, and 19.1% of the eyes had a UCVA ≥20/20, ≥20/40, and ≥20/80, respectively, which increased to 40.4%, 78.7%, and 93.6% at the 12 months postoperative examination, respectively. Also, in the preoperative examination, 68.1%, 80.9%, and 95.7% of the eyes had a BCVA ≥20/20, ≥20/40, and ≥20/80, respectively, which increased to 70.2%, 89.4%, and 97.9% at the 12 months postoperative examination, respectively. Notably, only in 1 eye (2.1%) postoperative BCVA was decreased 1 line of the Snellen chart (Figures 7 and 8).

**Table 6.** Comparison of pre and postoperative (12 months) UCVA and BCVA.

**Visual Acuity (LogMAR)**	**Mean±SD**	**Range**	**Mean diff±SD**	**P**
**UCVA**				
*Pre-op*	0.76±0.28	0.00–1.3	0.56±0.25	0.00
*Post-op*	0.19±0.22	0.00–0.78		
**BCVA**				
*Pre-op*	0.13±0.24	0.00–1.00	0.05±0.14	0.01
*Post-op*	0.08±0.16	0.00–0.7		

SD – Standard Deviation; UCVA – Uncorrected Distant Visual Acuity; BCVA – Best Corrected Distant Visual Acuity; diff – difference.

## Discussion

The present study evaluated 47 eyes from 25 patients with cycloplegic hyperopia of +1.00–+7.00 D and cycloplegic astigmatism ≤1 D. The mean and standard deviation of the preop cycloplegic sphere was +5.02±1.62 D, which significantly decreased to 1.83±1.72 at the 12 months postop.

Frings A *et al.* (2016) compared the effect of LASIK and PRK in the correction of hyperopia. The preoperative refractive sphere significantly decreased from +1.89±0.96 and +1.84±0.73 to 0.25±0.55 and 0.11±0.34 at the 6 months postop, respectively [19]. Also, in a similar study, Göker S *et al.* (1998) showed that the mean preoperative hyperopia decreased from +6.5±1.33 (+4.25–+8.00) to the mean sphere of +0.44±1.95 at 18 months after LASIK [17]. In a study by Alió JL (2013) preop cycloplegic sphere decreased from 6.33±0.83 with a range of 5–8.5 D to -0.32±0.61 with a range of -2.00–0.5 D at 6 months after the LASIK [2]. Many other studies confirmed the significant decrease of hyperopia using LASIK and PRK, which are in line with the data of the present study [4, 5, 7–10, 14, 15, 20].

In this study, the mean cycloplegic SE decreased from +4.76±1.55 with a range of 1.38–6.5 to +1.35±1.51 with a range of -1.00 to +5.38 at the 12-month postop.

Autrata R *et al.* (2003) showed that the mean SE before PRK decreased from +3.58±1.29 with a range of 1.75–7.50 D to 0.74±0.35 with a range of -0.5 to 1.75 D [18]. In the study by Frings A *et al.* (2016), the mean SE before the PRK decreased from +1.04±0.58 to -0.08±0.38 at the 6 months postop [19]. Kaluzny BJ *et al.* (2018) revealed that the mean SE decreased from +4.61±0.67 with a range of 3.6–6.15 D to -0.002±0.43 D at the 12 months postop [13]. Many other studies confirmed the significant decrease of the mean SE after PRK [2, 7–10, 12, 14, 15, 17, 20], which is in line with data from this study. However, the SE range at the 12 months postop in the present study was higher than in the other studies, so the postop residual or regressed SE was up to +5.38, which was not consistent with the previous studies. Of course, in previous studies as well, the higher the mean preop SE, the higher the mean postop SE, though it was lower than in our study.

In this study, 61.7% of the eyes had a cycloplegic sphere ≤2 D 12 months postoperatively. Also, 21.3%, 2.1%, and 15% of the eyes had cycloplegic spheres 2.25–3, 3.25–4, and >4 D, respectively.

According to Autrata R *et al.* [18], after 2 years of follow-up, 86% of patients in the PRK group showed an emmetropia of ±1.00 D interval and 57% SE of ±0.5 D in their eyes. In the studies by Kaluzny BJ *et al.* [13] and Yenugandula R *et al.* [21] (after 3 years), 78% and 92% of the eyes were in the range of ±0.5 and ±0.1.00 D, respectively. Also, in a study by El-Agha MS *et al.* [4], in the PRK group, after one year, 83.3% and 100% of the eyes had an SE of ±0.5 D and ±1.00 D, respectively, which was not in line with our results.

In a study by Wagh VK *et al.* [7], at the end of 18 years postop, 25% and 11% of the eyes had an SE of ±1.00 and ±0.5 D, respectively. In a study by Pietilä J *et al.* [5], in the low-moderate hyperopia group (<6 D), 26.7% and 50% of the eyes had hyperopia of 2.1–3 and 1.1–3 D at the 12 months postop, respectively. Also, in the high hyperopia group (≥6 D), 25% and 75% of the eyes had an SE of ≤2 D and >2 D, respectively, which was in line with the data from the present study.

Some studies had a more improved postop SE than others because of higher postop astigmatism than preop; therefore, in the present study, special emphasis is placed on separate evaluation and representation of cycloplegic sphere cycloplegic astigmatism and cycloplegic SE.

In this study, 17 eyes (36.2%) had astigmatism of >1 D at the 12 months postop, which decreased the mean postop SE (+1.35±1.51) compared to the postop mean cycloplegic sphere (+1.83±1.72). The mean preop cycloplegic cylinder significantly increased from -0.50±0.41 with a range of -1.00–0.00 D to -1.03±.88 with a range of -3.75–0.00 at 12 months postop. Llovet F *et al.* (2009) demonstrated that the preop cycloplegic cylinder in all patients decreased from -0.9±0.8 D to -0.3±0.4 at the 12 months postop [9]. Other studies also reported a decrease in the mean postop astigmatism, which was not consistent with our study [2, 12, 13, 16, 19, 20].

In the studies by Autrata R *et al.* [18] and El-Agha MS *et al.* [4], postop astigmatism increased slightly, and in other studies, there was a relatively high increase in postop astigmatism compared to preoperative ones [7, 10, 17], and the results of recent studies are in line with the data from the present study.

The postop induction of astigmatism received less attention in previous studies, whereas in the present study, despite preop astigmatism <1 D, there was astigmatism of >1 D with a range of 1.25–3.75 D in 17 eyes (36.2%) at the 12-month postop.

A significant association was observed between astigmatism >1 D at the 12 months postop with preop cycloplegic hyperopia >5 D, the postop residual cycloplegic hyperopia ≥2 D, and corneal ring haziness at the 12 months postop. Moreover, there was a significant association of 0.047 between the age ≤35 years and astigmatism >1 D postop, which is justified by the significantly high number of hyperopic >5 D (P=0.001) with ≤35 years of age compared to patients with >35 years of age.

There was stability in the cycloplegic sphere and SE at the 12 and 6 months postop, probably due to the simultaneous increase in astigmatism, making the SE changes less noticeable at the 6 months (0.43±0.46) compared to the cycloplegic sphere changes (0.52±0.51) in the same month.

In a study by Autrata R *et al.* [18], the SE in the PRK group reached stability at 9 months while O'Brart DP *et al.* [10], reported that stability of SE was obtained shortly after 12 months but continued until 7.5 years with +0.28 D. The results of the abovementioned studies were in line with the data achieved from this study.

The mean preop keratometry (Mean k) was 42.65±1.26 with a range of 39.50–45.31 D, which significantly increased to 45.48±2.13 with a range of 41.50–49.75 D at the 12 months postop. The increase in the mean keratometry in the first month was high, reaching 47.03±2.06, and then decreased to 46.40±1.97 and 45.87±2.02 D at the 3 and 6 months, respectively. This decrease in the mean keratometry was consistent with hyperopia regression.

In the study by Llovet F *et al.* [9], the mean preop keratometry significantly increased from 43.8±1.4 to 46.04±1.95 D 12 months postop, while in other studies [16, 19], the mean preop keratometry increased from 42.81±1.81 and 43.33±1.89 to 45.53±2.01 and 44.22±1.82 at 12 months postop, respectively. In our study, stability in the mean keratometry (<0.5 D) was obtained at 6 months postop, which was consistent with the study by Frings A *et al.* [19]. 

Corneal haziness around the optical zone was also studied, although late-onset haziness was particularly emphasized. None of the eyes had corneal haziness at the 1-month postop in this study. Furthermore, at the 3 months postop, corneal haziness was observed only in 5 eyes (10.6%). This number reached 25 eyes (53.2%) at the 6 and 12 months postop, although, at the 12 months, the number of eyes with grade 4 haziness was higher than at the 6 months postop. At the 12 months postop, the mean haziness was 2.34. In the study by Autrata R *et al.* [18], after 2 years of follow-up, in the PRK group, the mean epithelial haziness was 0.83±0.73. In the mentioned study, there was no association between haziness and recurrence rate that was inconsistent with the data from this study.

In a study by Kaluzny BJ *et al.* [13], 9.8% of the eyes had corneal haziness with a mean of 0.6 at the 12 months after PRK. Furthermore, another study reported that 25% and 70% of the eyes had haziness and iron ring 7 years after PRK [10]. The haziness and iron ring in the mentioned study were significantly associated with high preop hyperopia. In a study by Wagh VK *et al.* [7], 18 years after PRK, 40% and 9% of the eyes had corneal ring haziness and Salzmann nodular degeneration, respectively. Corneal haziness was also more prevalent in the high hyperopia group before surgery [5]. These results were in line with the data from our study, where the prevalence of haziness had a significant association with preop cycloplegic sphere >5 D and the residual cycloplegic hyperopia ≥2 D at the 12 months postop.

Also, a brown corneal iron ring was observed in 12 eyes (31.9%) at 12 months postop and did not have a significant association with preop cycloplegic hyperopia >5 D, which was not consistent with some of the previous studies [7, 10].

2.1%, 10.6%, and 19.1% of the eyes had a preoperative UCVA ≥20/20, ≥20/40, and ≥20/80, respectively, which increased to 40.4%, 78.7%, and 93.6% at the 12 months postop, respectively, indicating a significant improvement of UCVA.

In the study by Autrata R *et al.* [18], in the PRK group, after 2 years, 73% and 81% of the eyes had a UCVA ≥20/20 and ≥20/40, respectively. Preoperative UCVA in the PRK group increased from mean LogMAR of 0.37±0.28 to mean LogMAR of 0.03±0.11 at 6 months postop [19]. Furthermore, UCVA ≥20/20 was observed in 59% of eyes at the 3 years postop [13] and vision ≥20/40 was observed in 78.4% of the eyes at the 3 years postop [21]. In O'Brart DP *et al.* study [10], the UCVA ≥20/20 and ≥20/40 was observed in 15% and 57.5% of the eyes, respectively.

On the other hand, Pietil J *et al.* [5] reported no eyes with preop UCVA ≥20/40 in the high hyperopia group. At the 12 months postop, 8.3% and 41.7% of the eyes had UCVA ≥20/20 and ≥20/40, respectively. Also, El-Agha MS *et al.* [4] identified 41.7% and 63.2% of the eyes with a UCVA ≥20/20 and ≥20/40, in the PRK group, respectively. In the mentioned studies, there was a significant improvement in the postop uncorrected vision, which was in line with the data achieved from the present study.

In our study, 68.1%, 80.9%, and 95.7% of the eyes had a preop BCVA ≥20/20, ≥20/40, and ≥20/80, respectively, which increased to 70.2%, 89.4%, and 97.9% at the 12 months postop, respectively, which were consistent with other studies [4, 5, 7, 10, 13, 18, 22].

80.85% of eyes had an unchanged postop (12 months) BCVA compared to preoperation. In 2.12% of the eyes, 1-line loss of BCVA was observed at the 12 months postop. Also, in 4.25% and 12.78% of the eyes, 1 and 2 lines improvement was observed, respectively. None of the eyes had ≥2 lines of vision loss.

In Autrata R *et al.* study [18], in the PRK group, at the end of 2 years, no change was observed in 71% of the eyes in the postop BCVA. Also, 12% of the eyes had 1-line loss, and none of the eyes had ≥2 lines of vision loss. Furthermore, 11% and 6% of the eyes had an increased acuity as 1 and 2 lines of the Snellen chart than before the surgery. In other studies [5, 7, 13, 18], the results were similar to the mentioned study, consistent with the present study results.

## Conclusion

Despite the significant decrease of the postop sphere and SE, due to the relatively high number of eyes with cycloplegic hyperopia >2 D and/or astigmatism >1 D at the 12-month postop also, a relatively high level of corneal haziness and continued hyperopia regression (albeit as <0.5 D even until 12 months), PRK with Allegretto WaveLight Eye-Q 400 in people with cycloplegic hyperopia >5 D is not recommended. However, if the patient insists on performing PRK, a full explanation should be given to the patient.

## Acknowledgments 

### Conflict of Interest

The authors declare no conflict of interest. 

### Ethical approval

This study was approved by the Ethics Committee of Ardabil University of Medical Sciences (NO: IR.ARUMS.REC.1396.41) with an ethical code of IRCT NO: 20160318027097N9.

### Consent to participate

During the recruiting process, written informed consent was obtained from the participants for prospective data analysis.

### Authorship

HO contributed to conceptualizing, writing the original draft and data collection. FA contributed to the methodology. BS contributed to editing the manuscript and data curation. FA contributed to data analysis.
